# Cost-effectiveness analysis of immune checkpoint inhibitors combined with targeted therapy and chemotherapy for HPV/HIV-related cervical cancer

**DOI:** 10.1097/MD.0000000000040678

**Published:** 2024-11-29

**Authors:** Yuqing Liang, Aixia Ma

**Affiliations:** a Department of Pharmacoeconomics, School of International Pharmaceutical Business, China Pharmaceutical University, Nanjing, Jiangsu, China; b Center for Pharmacoeconomics and Outcomes Research, China Pharmaceutical University, Nanjing, Jiangsu, China.

**Keywords:** atezolizumab, cervical cancer, cost-effectiveness analysis, metastatic, pembrolizumab

## Abstract

**Background:**

To systematically assess the cost-effectiveness of immune checkpoint inhibitors compared to the current standard therapy for human papillomavirus (HPV) and human immunodeficiency virus (HIV)-related cervical cancer.

**Methods:**

A partitioned survival model spanning a 20-year period was created to evaluate the cost and effectiveness of atezolizumab combined with bevacizumab and chemotherapy (ABC), and pembrolizumab combined with bevacizumab and chemotherapy (PBC) vs bevacizumab combined with chemotherapy (BC), based on clinical data from the BEATcc and KEYNOTE-826 trials. Royston-Parmar models were used for survival estimation. Costs and health state utilities were sourced from existing literature and publicly accessible databases. Cumulative costs (in US dollars), life years, quality-adjusted life years (QALYs), and incremental cost-effectiveness ratios (ICERs) were measured and compared. The evaluation was from the US healthcare payer perspective, with the willingness-to-pay threshold set at $100,000 to $150,000. Deterministic sensitivity analysis (DSA), probabilistic sensitivity analysis (PSA), and scenario analyses were conducted.

**Results:**

The base-case analysis showed QALYs of 2.05 for BC, 3.18 for PBC, and 2.85 for ABC. PBC increased life-years by 1.76 and ABC by 1.18, with PBC showing the highest effectiveness. Total costs were $272,377 for BC, $715,472 for ABC, and $694,239 for PBC; severe adverse event (SAE) costs were $6189 for BC, $7603.31 for ABC, and $13,640 for PBC, indicating BC had the lowest SAE costs. The ICERs compared to BC were $372,151/QALY for PBC and $553,995/QALY for ABC. Given that the willingness-to-pay threshold was $100,000 to $150,000/QALY, both PBC and ABC exceed this threshold and were not considered cost-effective. BC had the lowest QALYs and the lowest costs, making it the least expensive option and the most cost-effective choice. DSA results indicated that drug prices and utility values were the main factors affecting cost-effectiveness. PSA confirmed BC as the most cost-effective option within a willingness-to-pay threshold of $0 to $300,000, primarily because it was the least costly.

**Conclusions:**

Immune checkpoint inhibitors significantly improve survival benefits for patients. However, their addition is costly and unlikely to be cost-effective for HPV/HIV-related metastatic cervical cancer.

## 1. Introduction

Cervical cancer remains a significant public health challenge, particularly in its metastatic form, which is associated with poor prognosis and limited treatment options.^[1]^ Traditional therapies often fail to provide satisfactory outcomes for patients with advanced cervical cancer, underscoring the urgent need for innovative treatments.^[2,3]^ The treatment landscape for advanced cervical cancer has significantly evolved. Historically, options were limited to surgery, radiation, and chemotherapy, which often proved inadequate for advanced stages. Recent advancements have introduced targeted therapies and immunotherapies, offering new hope. Bevacizumab, a VEGF inhibitor, has improved survival rates when combined with chemotherapy.^[4]^ Specifically, bevacizumab combined with chemotherapy regimens such as cisplatin and paclitaxel has become a standard therapy for advanced cervical cancer.^[5]^ Recent randomized controlled trials have further validated the effectiveness of these therapies. The KEYNOTE-826 trial demonstrated that pembrolizumab, when added to standard chemotherapy, significantly improved overall survival (OS) and progression-free survival (PFS) in patients with persistent, recurrent, or metastatic cervical cancer.^[6]^ Similarly, the BEATcc trial showed that the combination of atezolizumab with bevacizumab and chemotherapy led to improved outcomes in patients with advanced cervical cancer.^[7]^

However, the introduction of immune checkpoint inhibitors comes with substantial costs. The high prices of these therapies raise important questions about their overall cost-effectiveness, especially in the context of the United States, where healthcare costs are a major concern.^[8]^ The economic burden of cancer treatment is further exacerbated by declining incidence of AIDS, which can limit access to these potentially life-saving therapies for patients who need them the most.^[9]^ Evaluating the cost-effectiveness of immune checkpoint inhibitors is crucial to ensure that healthcare resources are used efficiently and that patients receive the best possible care without undue financial strain.^[10]^

Treating metastatic cervical cancer presents significant challenges, particularly when compounded by the intersecting burden of human immunodeficiency virus (HIV)/AIDS. This dual burden disproportionately impacts populations already vulnerable to cervical cancer.^[11]^ Women living with HIV face a higher risk of developing cervical cancer, primarily due to immunosuppression and an increased prevalence of human papillomavirus (HPV) infections.^[12]^

This study analyzed the cost-effectiveness of immune checkpoint inhibitors (atezolizumab and pembrolizumab) vs standard therapy for HPV/HIV-related persistent, recurrent, or metastatic (stage ⅣB) cervical cancer, aiming to inform healthcare decisions and enhance treatment access and outcomes in the United States.

## 2. Materials and methods

### 2.1. Patients and interventions

This analysis evaluated cost-effectiveness by utilizing a literature review and modeling techniques. Through the literature review, we identified relevant model parameters, including clinical parameters, cost parameters, safety parameters, resource utilization parameters, and utility values. The target population was based on the BEATcc^[7]^ and KEYNOTE-826^[6]^ trial populations: adults aged 18 and older with persistent, recurrent, or metastatic cervical cancer who had not received systemic chemotherapy and were not candidates for curative treatment.

Three treatment regimens were evaluated: bevacizumab combined with chemotherapy (BC), atezolizumab combined with bevacizumab and chemotherapy (ABC), and pembrolizumab combined with bevacizumab and chemotherapy (PBC). Based on the BEATcc and KEYNOTE-826 trials, the specific medications for these regimens are as follows: BC involves administering bevacizumab intravenously at a dose of 15 mg/kg every 3 weeks, with chemotherapy typically including paclitaxel and cisplatin or carboplatin, administered in 6 cycles, each cycle lasting 21 days. ABC includes atezolizumab at 1200 mg every 3 weeks and bevacizumab at 15 mg/kg every 3 weeks, along with similar chemotherapy regimens administered in 6 cycles of 21 days each. For atezolizumab, the base-case analysis assumed a maximum usage of 2 years. However, in scenario analyses, continued use beyond 2 years was allowed to maintain consistency with the BEATcc trial. PBC involves pembrolizumab at 200 mg every 21-day cycle for up to 35 cycles and bevacizumab at 15 mg/kg every 3 weeks, also paired with paclitaxel and cisplatin or carboplatin, administered in 6 cycles of 21 days each. Following progression, the most commonly used approaches were best supportive care (BSC) and monotherapy chemotherapy.^[7]^

### 2.2. Model overview

**Figure 1.**
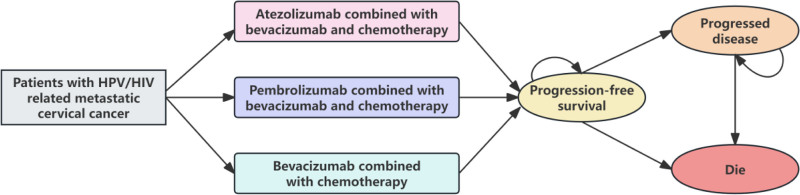
Model structure plot.

A partitioned survival model (Fig. 1) consisting of 3 health states (PFS, progressed disease [PD], and death) was created using Microsoft Excel (2019) to evaluate the costs and clinical outcomes of the 3 treatment regimens for a cohort of women with persistent, recurrent, or metastatic cervical cancer. In this model, the proportion of patients in each health state at different time points was directly obtained from the PFS and OS curves.^[13]^ The model had a cycle length of 3 weeks and a life-time horizon of 20 years. This analysis was performed from the perspective of US healthcare payers. It measured life-years, quality-adjusted life-years (QALYs), overall costs, and the incremental cost-effectiveness ratios (ICERs) between treatments.^[14]^ A 3% annual discount rate was applied to both costs and effectiveness, as recommended in the US.^[15]^ The willingness-to-pay (WTP) threshold was set between $100,000 and $150,000 per QALY.^[10]^

### 2.3. Clinical data inputs

OS and PFS probabilities for the BC and ABC regimens were obtained from Kaplan–Meier (KM) curves in the BEATcc trial using GetData Graph Digitizer (http://getdata-graph-digitizer.com) and the methodology described by Guyot to reconstruct individual patient data (IPD) estimates over the clinical trial duration.^[16]^ Survival rates for the PBC regimen were derived indirectly. Since the use of bevacizumab in the KEYNOTE-826 trial was selective (determined by the investigator), the overall population survival curves did not fully apply to the PBC regimen we studied. Additionally, the KM curves for the subgroup that received bevacizumab were not publicly available. Therefore, we could only use the hazards ratios (HRs) for the PBC vs BC regimens based on the subgroup that received bevacizumab in the KEYNOTE-826 trial and anchored these to the BC curves from the BEATcc trial to calculate the survival rates for each cycle of the PBC regimen.^[6,7]^ The virtual IPD, which included event and censor times, closely matched the initial at-risk population, accurately replicating the digitized KM curves. These IPD were then used to fit various survival models: exponential, Weibull, Gompertz, gamma, log-logistic, log-normal, generalized gamma, fractional polynomial, restricted cubic spline models, and Royston–Parmar spline models. Model fit was evaluated using the Akaike information criterion (AIC) and visual examination.^[17,18]^ Overall, the Royston–Parmar spline models with hazards and odds as baseline link functions was selected. The fit of the parametric models and the final chosen model are detailed in Table S1, Supplemental Digital Content, http://links.lww.com/MD/O53 and Figure S1, Supplemental Digital Content, http://links.lww.com/MD/O52.

Additionally, the impact of grade 3 or higher adverse events (SAEs) with an incidence of over 5% was included in the analysis, with data sourced from the BEATcc and KEYNOTE-826 trials.

### 2.4. Costs and utilities

Direct medical costs encompassed expenses related to medications, administration, follow-up, management of SAEs, best supportive care, and end-of-life care. The unit prices were obtained from the Centers for Medicare & Medicaid Services.^[19,20]^ The costs for SAE management, best supportive care, and end-of-life care were estimated from published literature. All costs were adjusted to 2024 values using the US consumer price index.^[21]^ Utility values were sourced from a published cost-effectiveness analysis for the same indication.^[22]^ The disutility of 0.28 for SAEs was obtained from the literature, and were assumed to occur in the first cycle.^[22,23]^ The values and sources of all parameters are detailed in Table 1.

**Table 1:** Basic parameters input to the model and the ranges for sensitivity analyses.

Parameters	Mean	SD	Range	Distribution	Source
Cost in the US (US dollar $)					
Cost_best supportive care	3835.0	442.8	3068–4602	Gamma	^[22]^
Cost_end-of-life care	12,797.9	1477.8	10,238–15,358	Gamma	^[22]^
Follow-up cost	547.86	63.3	438–657	Gamma	^[24]^
Administration cost	174.0	20.1	139–209	Gamma	^[25]^
Cost_paclitaxel_1 mg	0.1	0	0.08–0.12	Gamma	^[19]^
Cost_bevacizumab_10 mg	72.5	8.4	58–87	Gamma	^[19]^
Cost_cisplatin_10 mg	3.7	0.4	3–5	Gamma	^[19]^
Cost_carboplatin_50 mg	3.7	0.4	3–4	Gamma	^[19]^
Cost_pembrolizumab_1 mg	56.7	6.5	45–68	Gamma	^[19]^
Cost_atezolizumab_10 mg	85.8	9.9	69–103	Gamma	^[19]^
Cost_peripheral or sensory neuropathy	9227.1	1065.4	7382–11,072	Gamma	^[26]^
Cost_asthenia	9860.3	1138.5	7888–11,832	Gamma	^[26]^
Cost_anaemia	15,960.2	1842.9	12,768–19,152	Gamma	^[22]^
Cost_neutropenia	11,562.1	1335.1	9250–13,874	Gamma	^[22]^
Cost_hypertension	2814.5	324.9	2251–3377	Gamma	^[22]^
Cost_proteinuria	5799.3	669.6	4639–6959	Gamma	^[27]^
Cost_thrombocytopenia	14,056.1	1623.0	11,245–16,867	Gamma	^[22]^
Cost_urinary tract infection	9149.0	1056.4	7319–10,979	Gamma	^[22]^
Cost_platelet count decreased	15,954.1	1842.2	12,763–19,145	Gamma	^[26]^
Cost_white blood cell count decreased	10,782.8	1245.0	8626–12,938	Gamma	^[27]^
Cost_neutrophil count decreased	16,625.7	1919.7	13,300–19,950	Gamma	^[26]^
Cost_febrile neutropenia	17,597.1	2031.9	14,078–21,116	Gamma	^[28]^
Subsequent active treatment cost	208.9	1.3	207–211	Gamma	Calculated
Utility and disutility					
Utility_PFS	0.817	0.1	0.65–0.98	Beta	^[22]^
Utility_PD	0.779	0.1	0.62–0.93	Beta	^[22]^
Disu_SAE	0.280	0	0.22–0.34	Beta	^[22]^
Clinical data					
SAE_ABC_peripheral or sensory neuropathy	0.07	0	0.06–0.08	Beta	^[7]^
SAE_ABC_asthenia	0.11	0	0.09–0.13	Beta	^[7]^
SAE_ABC_anaemia	0.14	0	0.11–0.17	Beta	^[7]^
SAE_ABC_neutropenia	0.18	0	0.14–0.22	Beta	^[7]^
SAE_ABC_hypertension	0.18	0	0.14–0.22	Beta	^[7]^
SAE_ABC_proteinuria	0.06	0	0.05–0.07	Beta	^[7]^
SAE_ABC_thrombocytopenia	0.05	0	0.04–0.06	Beta	^[7]^
SAE_BC_asthenia	0.09	0	0.07–0.11	Beta	^[7]^
SAE_BC_anaemia	0.07	0	0.06–0.08	Beta	^[7]^
SAE_BC_neutropenia	0.25	0	0.2–0.3	Beta	^[7]^
SAE_BC_hypertension	0.16	0	0.13–0.19	Beta	^[7]^
SAE_BC_thrombocytopenia	0.06	0	0.05–0.07	Beta	^[7]^
SAE_PBC_anaemia	0.303	0	0.24–0.36	Beta	^[6]^
SAE_PBC_neutropenia	0.124	0	0.1–0.15	Beta	^[6]^
SAE_PBC_hypertension	0.094	0	0.08–0.11	Beta	^[6]^
SAE_PBC_thrombocytopenia	0.075	0	0.06–0.09	Beta	^[6]^
SAE_PBC_urinary tract infection	0.088	0	0.07–0.11	Beta	^[6]^
SAE_PBC_platelet count decreased	0.068	0	0.05–0.08	Beta	^[6]^
SAE_PBC_white blood cell count decreased	0.068	0	0.05–0.08	Beta	^[6]^
SAE_PBC_neutrophil count decreased	0.13	0	0.1–0.16	Beta	^[6]^
SAE_PBC_febrile neutropenia	0.072	0	0.06–0.09	Beta	^[6]^
SAE_ABC	0.79	0.1	0.63–0.95	Beta	^[7]^
SAE_BC	0.75	0.1	0.6–0.9	Beta	^[7]^
SAE_PBC	0.818	0.1	0.65–0.98	Beta	^[6]^
HR_OS_PEM VS C	0.63	0.1	0.47–0.87	Log-normal	^[6]^
HR_PFS_PEM VS C	0.61	0.1	0.47–0.79	Log-normal	^[6]^
Proportion of patients receiving subsequent active treatment after progression					
Subsequent active treatment_ABC	0.54	0.1	0.43–0.65	Beta	^[7]^
Subsequent active treatment_PBC	0.54	0.1	0.43–0.65	Beta	^[7]^
Subsequent active treatment_BC	0.58	0.1	0.46–0.7	Beta	^[7]^
Others					
Average weight of the US female	77.5			Fixed	^[22]^
Average body surface of the US female	1.86			Fixed	^[22]^
Discount	0.03	0	0–0.08	Beta	^[26]^

ABC = atezolizumab combined with bevacizumab and chemotherapy, BC = bevacizumab combined with chemotherapy, HR = hazard ratio, PBC = pembrolizumab combined with bevacizumab and chemotherapy, PD = progressed disease, PFS = progreesion-free survival, SAE = grade 3 or higher adverse events.

### 2.5. Cost-effectiveness analysis

We performed sensitivity analyses to evaluate the stability of the base-case results. For the deterministic sensitivity analysis (DSA), parameters were varied within their 95% confidence intervals or set to plausible ranges (±20% of the base-case values). Tornado diagrams illustrated the impact of changing individual parameters. Probabilistic sensitivity analysis (PSA) involved conducting a Monte Carlo simulation with 10,000 iterations. We used a gamma distribution for costs, a beta distribution for probabilities, proportions, and utilities, and a lognormal distribution for HR parameters. Cost-effectiveness acceptability curves depicted the cost-effectiveness of each regimen across different WTP thresholds. More details, see Table 1.

### 2.6. Ethical review

It is not necessary to obtain ethical approval for this study as it is based entirely on publicly available data.

## 3. Results

The base-case analysis results are presented in Table 2. The costs for the BC, PBC, and ABC regimens were $272,377, $694,239, and $715,472, respectively. The effectiveness for BC, PBC, and ABC was 2.05, 3.18, and 2.85 QALYs, respectively. Compared to BC, the ICERs for PBC and ABC were $372,151/QALY and $553,995/QALY, respectively. PBC was the dominant strategy over ABC. Using life-years as the outcome, compared to the standard therapy, the PBC and ABC regimens increased patient life-years by 1.76 and 1.18 years, respectively. In terms of safety, the costs due to SAEs for the BC, ABC, and PBC regimens were $6189, $7603, and $13,640, respectively. Thus, the BC and ABC regimens demonstrated better overall safety profiles compared to the PBC regimen, with BC incurring the lowest SAE costs.

**Table 2:** Results of base case analysis.

Treatment	Cost ($)	Effectiveness (QALY)	Life-year (yr)	VS. BC		
				Differences in costs	Differences in utilities	ICER ($/QALY)
BC	272,377	2.05	2.76	–	–	–
ABC	715,472	2.85	3.93	443,094	0.800	553,995
PBC	694,239	3.18	4.51	421,862	1.134	372,151

ABC = atezolizumab combined with bevacizumab and chemotherapy, BC = bevacizumab combined with chemotherapy, ICER = incremental cost-effectiveness ratio, PBC = pembrolizumab combined with bevacizumab and chemotherapy, QALY = quality-adjusted life-year.

### 3.1. Sensitivity analysis

**Figure 2.**
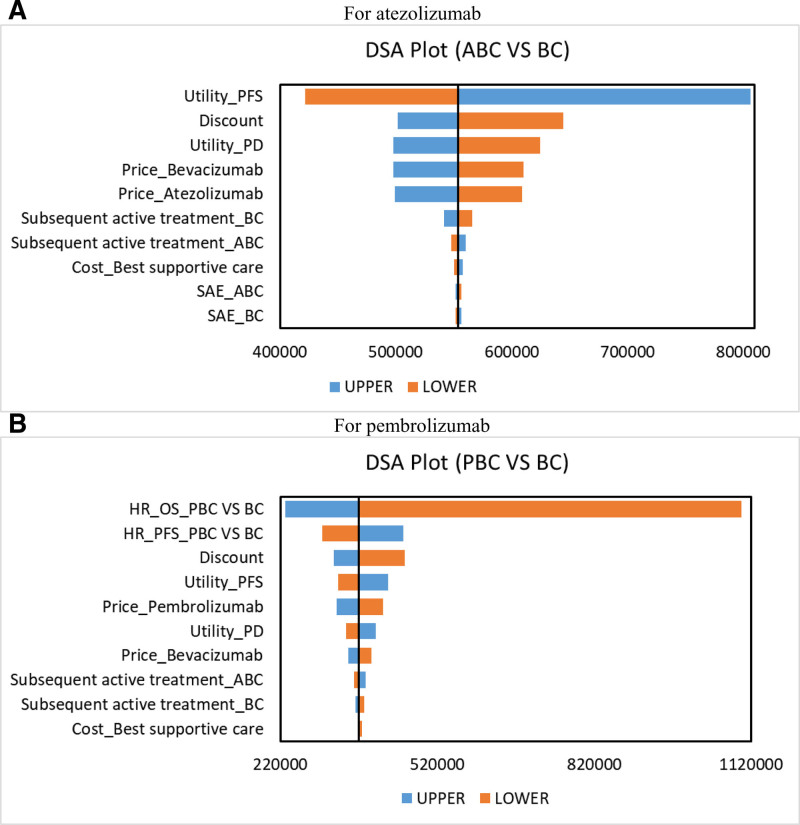
Tornado diagram for deterministic sensitivity analysis.

The DSA results showed that, in terms of ICER compared to the BC regimen, the factors influencing the cost-effectiveness of the PBC regimen, in descending order, were the HR values for OS and PFS of PBC compared to BC, the utility values of PFS and PD, and the prices of bevacizumab and pembrolizumab. However, even when these parameters fluctuated within their upper and lower limits, the PBC regimen compared to BC was never cost-effective, with ICER values consistently exceeding the WTP threshold of $150,000/QALY. Similarly, the main factors affecting the cost-effectiveness of the ABC regimen compared to BC were the utility values for PFS and PD states, and the prices of bevacizumab and atezolizumab. Nevertheless, under all parameter variations within the given range, the ICER of ABC compared to BC always significantly exceeded the upper limit of the WTP threshold. More details are provided in Figure 2.

**Figure 3.**
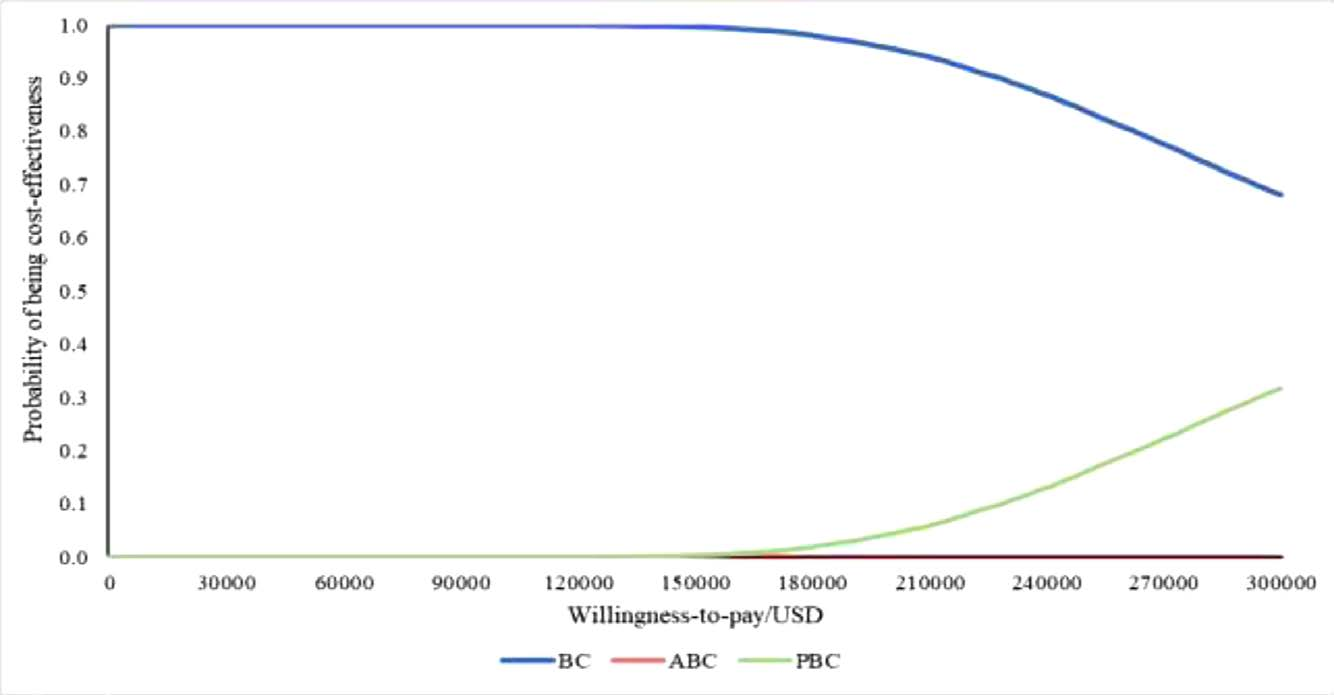
Cost-effectiveness acceptability curve plot.

The PSA results further validated the robustness of the base-case analysis conclusions. As shown by the cost-effectiveness acceptability curve (Fig. 3), when the WTP threshold ranged from $0 to $300,000, BC regimen consistently had the highest probability of being the lowest cost per QALY gained option. Specifically, within the WTP threshold range set for this study, the probability of the BC regimen being the most economical among BC, PBC, and ABC was consistently 100%.

The sensitivity analysis results indicated that the base-case conclusions were robust. Although the BC regimen was not the most effective, it remained significantly more cost-effective compared to the more effective PBC and ABC regimens. Between PBC and ABC, the PBC regimen was the superior choice in terms of both effectiveness and cost-effectiveness. Immune checkpoint inhibitors combined with targeted therapy and chemotherapy are not yet cost-effective compared to the current standard therapy.

### 3.2. Scenario analysis

When continued use of atezolizumab beyond 2 years was allowed, the cost and effectiveness of the atezolizumab regimen were $997,075 and 2.849 QALYs, respectively. Compared to BC, the ICER further increased to $906,079/QALY. It remains a significantly inferior option compared to PBC.

When the simulation upper limit was set to 10 years, the BC regimen cost $267,057 with an effectiveness of 1.959 QALYs. The ABC regimen cost $655,543 with 2.543 QALYs, resulting in an ICER of $665,232/QALY compared to BC. The PBC regimen cost $664,789 with 2.794 QALYs, resulting in an ICER of $476,478/QALY compared to BC. Compared to ABC, PBC had an ICER of $36,874/QALY, demonstrating a significant cost-effectiveness advantage.

When the simulation time was set to 5 years, the BC regimen cost $248,463 with an effectiveness of 1.733 QALYs and 2.755 life-years. The ABC regimen cost $565,653 with 2.080 QALYs and 3.934 life-years, resulting in an ICER of $913,575/QALY compared to BC. The PBC regimen cost $599,556 with 2.191 QALYs and 4.513 life-years, resulting in an ICER of $765,398/QALY compared to BC. Compared to ABC, PBC had an ICER of $351,093/QALY, showing a somewhat more favorable cost-effectiveness profile than ABC.

## 4. Discussion

The base-case analysis showed effectiveness values of 2.05, 3.18, and 2.85 QALYs for BC, PBC, and ABC, respectively. Using life-years as the outcome, PBC and ABC increased patient life-years by 1.76 and 1.18 years, respectively, with PBC demonstrating the best effectiveness. In terms of safety, the costs due to SAEs for BC, ABC, and PBC were $6188.70, $7603.31, and $13,639.68, respectively, indicating that BC had the best safety profile. From the cost-effectiveness perspective, compared to BC, the ICERs for PBC and ABC were $372,151/QALY and $553,995/QALY, with PBC being the dominant strategy over ABC and BC being the most cost-effective choice. Sensitivity analysis confirmed that factors such as HR values, utility values, and drug prices influenced the cost-effectiveness of PBC and ABC, but neither regimen was cost-effective compared to BC. PSA results showed that BC had the highest probability of being the most cost-effective option within a WTP threshold of $0 to $300,000. Overall, immune checkpoint inhibitors combined with targeted therapy and chemotherapy are not yet cost-effective compared to the current standard of care.

Several articles have evaluated the cost-effectiveness of systemic therapies for metastatic cervical cancer. Minion et al^[29]^ assessed the cost-effectiveness of bevacizumab for advanced cervical cancer using a Markov model based on data from the Gynecologic Oncology Group 240 trial.^[30]^ The model simulated patient transitions through various health states over 60 months, including response, progression, complications, and death. The study found that the high therapy cost was mainly due to the drug itself rather than the management of complications. It suggested that less expensive biosimilars could significantly reduce the ICER, making bevacizumab more viable for advanced cervical cancer treatment. Barrington et al^[31]^ evaluated the cost-effectiveness of pembrolizumab plus chemotherapy (PC) and PBC compared to BC in recurrent or metastatic cervical cancer using data from KEYNOTE-826. They calculated ICERs with a $100,000/QALY threshold and found PC to be cost-effective relative to BC, while PBC was not unless the cost of pembrolizumab decreased significantly. PBC’s efficacy would have needed to far exceed both BC and PC to be cost-effective. Shi et al^[22]^ evaluated the cost-effectiveness of pembrolizumab for treating persistent, recurrent, or metastatic cervical cancer in the US. Using a partitioned survival model and data from the KEYNOTE-826 trial, they found that pembrolizumab provided an additional 0.74 QALY at an incremental cost of $182,271 compared to placebo, resulting in an ICER of $247,663/QALY. The study concluded that pembrolizumab was unlikely to be cost-effective at its current price under the US healthcare system’s willingness-to-pay threshold.

The findings of the above study aligned with our conclusion that PBC was not cost-effective compared to BC. Our study built on this by systematically comparing the cost-effectiveness of all immune checkpoint inhibitors in metastatic cervical cancer. Additionally, we were the first to evaluate the economic value of ABC. Besides cost-effectiveness, we also analyzed differences in effectiveness and safety between immune checkpoint inhibitors and standard therapy. We used a more flexible model for survival analysis and conducted multiple uncertainty analyses, which enhanced our confidence in the conclusions.

Additionally, this study had certain limitations. Firstly, we lacked individual patient data from the BEATcc and KEYNOTE-826 trials, preventing us from conducting rigorous subgroup analyses. Similarly, the Kaplan–Meier curves for patients receiving bevacizumab in the KEYNOTE-826 trial were unavailable, so we only considered the Cox proportional hazards model when synthesizing PBC evidence. Due to the lack of reporting in BEATcc and KEYNOTE-826, the utility values and survival data were not homogenous, which might introduce some heterogeneity.

## 5. Conclusion

Immune checkpoint inhibitors significantly improve survival benefits for patients. However, their addition is costly and unlikely to be cost-effective for HPV/HIV-related metastatic cervical cancer compared to current standard therapy from the US healthcare payer perspective. Pembrolizumab, when combined with standard therapy, offers better survival benefits and an absolute cost-effectiveness advantage over atezolizumab. Meanwhile, the BC regimen had the lowest SAE costs and remained the least costly option.

## Author contributions

**Conceptualization:** Aixia Ma.

**Data curation:** Yuqing Liang, Aixia Ma.

**Formal analysis:** Yuqing Liang, Aixia Ma.

**Investigation:** Yuqing Liang, Aixia Ma.

**Methodology:** Yuqing Liang, Aixia Ma.

**Project administration:** Yuqing Liang, Aixia Ma.

**Resources:** Yuqing Liang, Aixia Ma.

**Software:** Yuqing Liang, Aixia Ma.

**Supervision:** Yuqing Liang, Aixia Ma.

**Validation:** Yuqing Liang, Aixia Ma.

**Visualization:** Yuqing Liang, Aixia Ma.

**Writing – original draft:** Yuqing Liang, Aixia Ma.

**Writing – review & editing:** Yuqing Liang, Aixia Ma.

## Supplementary Material


