# Supplementary material for: Cost-effectiveness analysis of immune checkpoint inhibitors combined with targeted therapy and chemotherapy for HPV/HIV-related cervical cancer
Source: Medicine (Baltimore). 2024 Nov 29;103(48):e40678. doi: 10.1097/MD.0000000000040678 (PMC11608711; doi:10.1097/MD.0000000000040678)
Supplement: Supplementary file 1 [file medi-103-e40678-s001.docx]

## **Supplement**

## Table S1 Goodness-of-fit results

| OS for ABC | | PFS for ABC | | OS for BC | | PFS for BC | |
| --- | --- | --- | --- | --- | --- | --- | --- |
| Model | AIC | Model | AIC | Model | AIC | Model | AIC |
| RP-odds-1 | 153.6332916 | RP-hazard-1 | 197.64741 | RP-odds-1 | 131.6260088 | RP-hazard-1 | 231.3716717 |
| llogis | 153.6332917 | RCS2 | 198.8011994 | FP1-1 | 132.4461751 | genf | 231.5240626 |
| gamma | 154.4490967 | RP-odds-1 | 198.1742957 | FP1-2 | 132.9280905 | RP-hazard-2 | 231.9954841 |
| RP-hazard-1 | 155.1923158 | RP-normal-1 | 198.8506376 | llogis | 133.6260087 | RP-normal-1 | 232.3655708 |
| weibull | 155.1923159 | RP-odds-2 | 199.3834076 | gamma | 133.6617491 | RP-normal-2 | 232.4599872 |
| RP-normal-1 | 155.2253237 | RP-hazard-2 | 200.0286284 | FP2-2 | 134.5500593 | RP-odds-1 | 232.6432146 |
| RP-odds-2 | 155.4491457 | FP2-1 | 204.3514975 | FP2-1 | 134.6858077 | RCS2 | 233.0265361 |
| RCS1 | 155.9196779 | FP2-2 | 208.6035137 | RP-normal-2 | 134.7314139 | RP-odds-2 | 233.0391664 |
| FP2-1 | 156.0632588 | RP-normal-2 | 218.458491 | lnorm | 134.7314143 | llogis | 239.452367 |
| RP-normal-2 | 156.2018128 | lnorm | 218.8701042 | RP-hazard-1 | 135.0329995 | FP2-1 | 246.4231249 |
| RCS2 | 156.3086549 | gengamma | 219.5735275 | weibull | 135.0329996 | lnorm | 248.7697781 |
| gengamma | 156.4416669 | RCS1 | 220.011758 | RP-normal-1 | 135.1957824 | FP2-2 | 248.960028 |
| FP2-2 | 156.6666001 | llogis | 220.1667235 | gengamma | 135.3719629 | gengamma | 249.9703874 |
| FP1-1 | 156.7054129 | FP1-2 | 235.5986262 | RP-hazard-2 | 135.5560932 | gamma | 261.8767015 |
| RP-hazard-2 | 156.7922785 | FP1-1 | 239.1635542 | RP-odds-2 | 135.6584759 | FP1-2 | 262.3463577 |
| genf | 157.3458405 | gamma | 240.7679648 | genf | 137.3760005 | FP1-1 | 264.4954373 |
| lnorm | 160.210327 | weibull | 244.624959 | RCS1 | 138.5851171 | RCS1 | 268.2163358 |
| gompertz | 162.2397695 | exp | 245.3744586 | RCS2 | 138.5965702 | weibull | 270.9842154 |
| FP1-2 | 163.7200992 | gompertz | 245.6382736 | gompertz | 143.9581438 | exp | 285.2012821 |
| exp | 171.3325678 |  |  | exp | 156.4019957 | gompertz | 285.7691669 |

Note: ABC, atezolizumab combined with bevacizumab and chemotherapy; BC, bevacizumab and chemotherapy; OS: overall survival; PFS: progression-free survival; LnL: log likelihood; Params: Parameters; AIC: Akaike information criterion; Exp: exponential; lnorm: log normal; llogis: log logistic; gengamma: generalized gamma; RP: Royston-Parmar models
