# Supplementary material for: Cost-effectiveness analysis of immune checkpoint inhibitors combined with targeted therapy and chemotherapy for HPV/HIV-related cervical cancer
Source: Medicine (Baltimore). 2024 Nov 29;103(48):e40678. doi: 10.1097/MD.0000000000040678 (PMC11608711; doi:10.1097/MD.0000000000040678)

## **Supplement**

## Figure S1 Survival plots showing the goodness-of-fit

Overall survival for atezolizumab combined with bevacizumab and chemotherapy


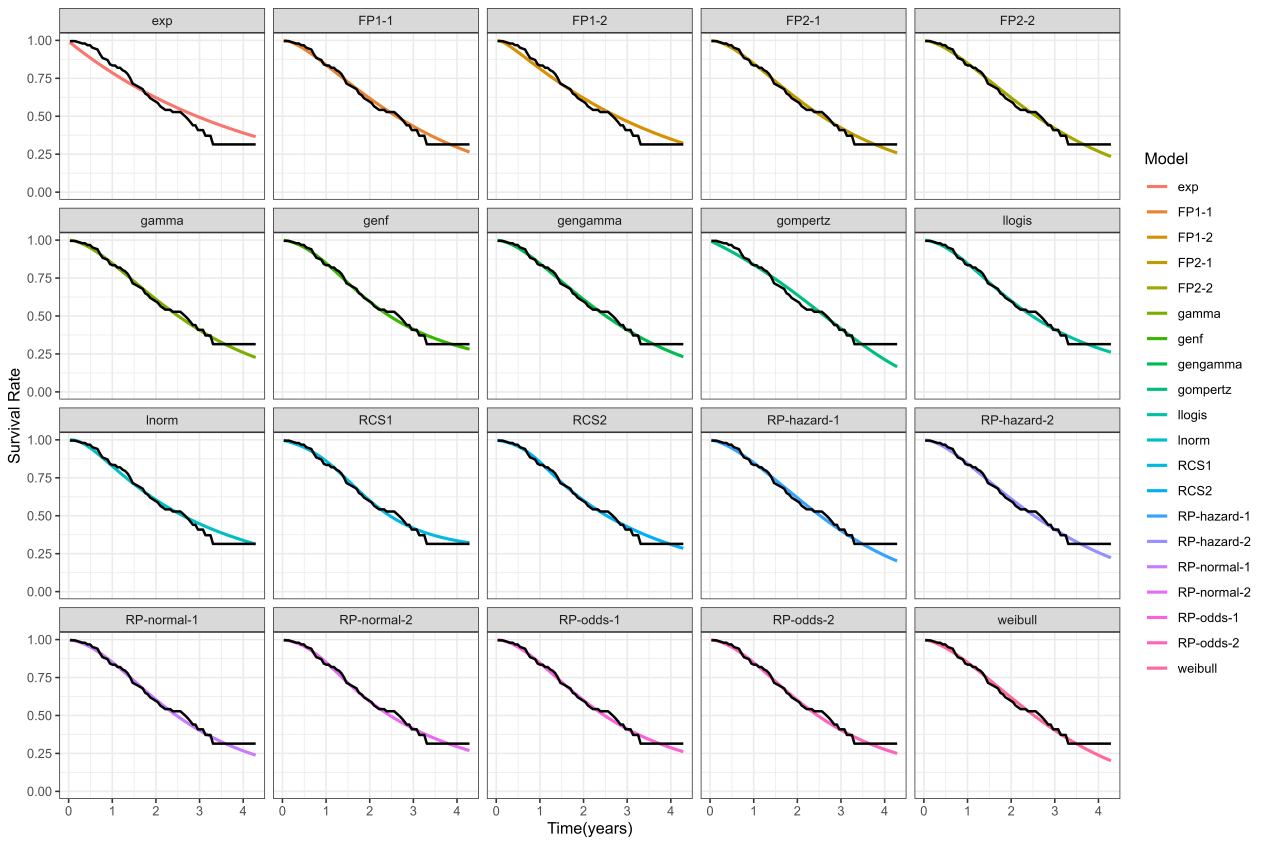


Overall survival for bevacizumab combined with chemotherapy





Progression-free survival for atezolizumab combined with bevacizumab and chemotherapy





Progression-free survival for bevacizumab combined with chemotherapy


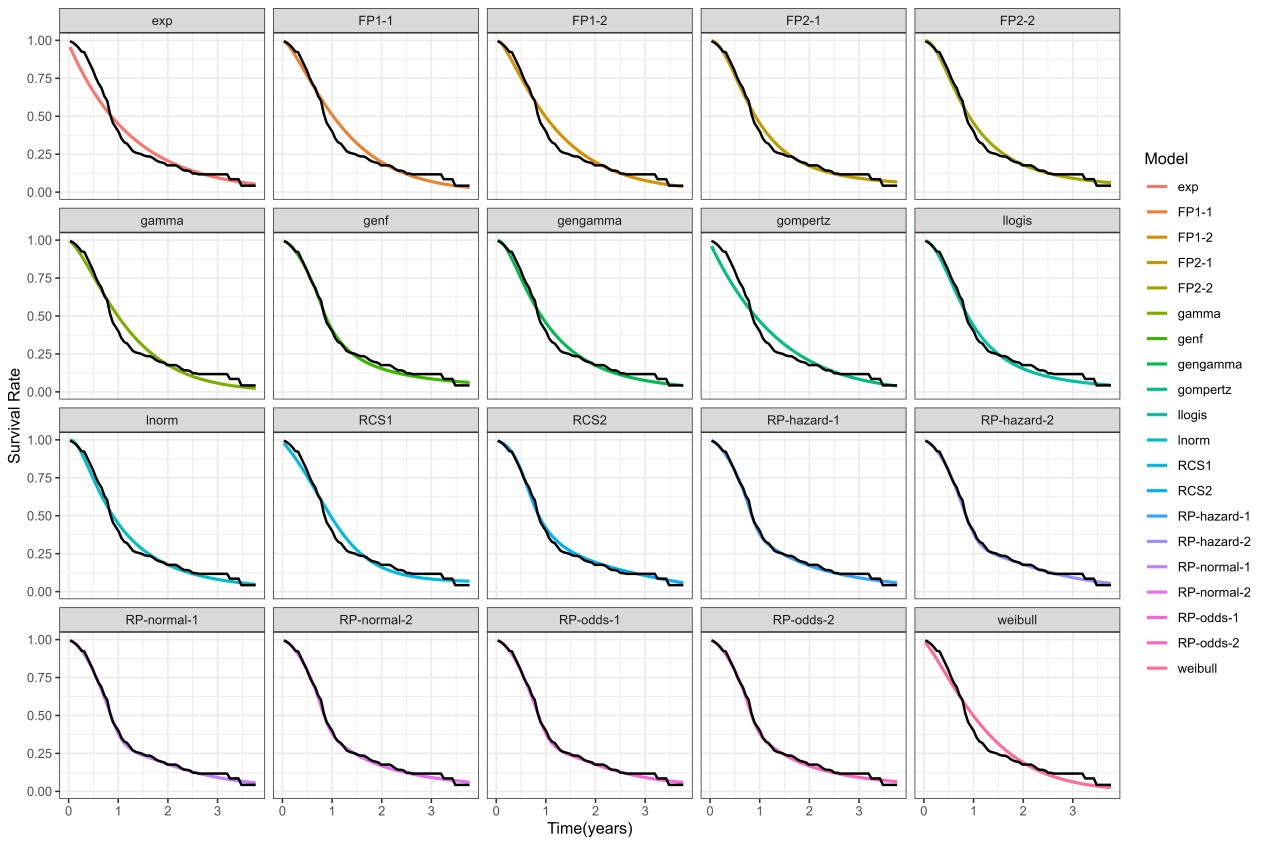

Supplement: Supplementary file 2 [file medi-103-e40678-s002.docx]
